# ATM Protein Kinase: Old and New Implications in Neuronal Pathways and Brain Circuitry

**DOI:** 10.3390/cells9091969

**Published:** 2020-08-26

**Authors:** Lara Pizzamiglio, Elisa Focchi, Flavia Antonucci

**Affiliations:** 1 Institute of Molecular and Cellular Pharmacology (IPMC), Université Côte d’Azur (UCA), CNRS UMR7275, 06560 Valbonne-Sophia Antipolis, France; pizzamiglio@ipmc.cnrs.fr; 2 Department of Medical Biotechnology and Translational Medicine (BIOMETRA), University of Milan, 20100 Milan, Italy; elisa.focchi@unimi.it

**Keywords:** neurons, neurodegeneration, brain circuits, cell signalling

## Abstract

Despite that the human autosomal recessive disease ataxia telangiectasia (A-T) is a rare pathology, interest in the function of ataxia-telangiectasia mutated protein (ATM) is extensive. From a clinical point of view, the role of ATM in the central nervous system (CNS) is the most impacting, as motor disability is the predominant symptom affecting A-T patients. Coherently, spino-cerebellar neurodegeneration is the principal hallmark of A-T and other CNS regions such as dentate and olivary nuclei and brain stem are implicated in A-T pathophysiology. Recently, several preclinical studies also highlighted the involvement of ATM in the cerebral cortex and hippocampus, thus extending A-T symptomatology to new brain areas and pathways. Here, we review old and recent evidence that largely demonstrates not only the historical ATM account in DNA damage response and cell cycle regulation, but the multiple pathways through which ATM controls oxidative stress homeostasis, insulin signalling pathways, epigenetic regulation, synaptic transmission, and excitatory–inhibitory balance. We also summarise recent evidence on ATM implication in neurological and cognitive diseases beyond A-T, bringing out ATM as new pathological substrate and potential therapeutic target.

## 1. Introduction

ATM (ataxia-telangiectasia mutated) belongs to a family of large phosphoprotein kinases found in various organisms and ubiquitously expressed. Functional inactivation of the *ATM* gene product accounts for a complex disorder with a highly complex phenotype called ataxia telangiectasia (A-T) [1], whose estimated prevalence is of 1:40,000 to 1:100,000 [2]. It was in 1988 that, using genetic linkage analysis of 31 A-T families, Gatti and colleagues first localized candidate gene(s) for A-T in chromosomal region 11q22-23 [3]. However, it was not until 1995 that one single gene was identified as the cause of the disease and subsequently named *ATM* (A-T mutated) [4]. This gene occupies 160 kb of the entire genomic DNA and encodes a 12 kb transcript of 66 exons [5].

Most human *ATM* mutations lead to the destabilization of the message, the protein, or both [6,7], resulting in A-T neurodegenerative pathology, mainly characterized by progressive cerebellar ataxia and oculocutaneous telangiectasia [8]. A-T symptoms correlate with the degree of ATM serine/threonine protein kinase inactivation. In fact, in classic A-T, the phenotype is determined by homozygosity or compound heterozygosity for *ATM* alleles, with no protein produced and kinase activity totally absent [9]. Milder forms of A-T are characterized by later onset and slower progression owing to missense rather than truncating mutations or promoter defects, which lead to the production of reduced amounts (between 1% and 17%) of functional ATM protein [10]. Therefore, A-T variants provide a genotype/phenotype relationship for understanding how specific *ATM* mutations contribute to the disease [11].

A-T generally appears early in childhood. Patients start displaying dyssynergia (abnormal movements of the body), muscle hypotonia, truncal swaying while sitting or standing, and sudden falls [11], up to having trouble walking independently around the age of three [12] and starting to use a wheelchair by adolescence. The principal hallmark of A-T is spino-cerebellar neurodegeneration as confirmed by the (i) progressive and massive cortical cerebellar degeneration related mostly to Purkinje and granule cells [13] and (ii) cerebellum atrophy, evident upon magnetic resonance and computed tomography imaging [14]. As cerebellar granule cells account for the majority of neurons in the human brain, they profoundly affect cerebellar function. Moreover, Purkinje neurons show a simplified dendritic arborizations and are often localized ectopically in the molecular layer of the cerebellum [15]. Their abnormal localization also suggests an early developmental defect in A-T, as the spatiotemporal distribution of Purkinje cells occurs relatively early during nervous system development [16]. Widespread neurodegeneration also occurs in other CNS regions, such as dentate and olivary nuclei, basal ganglia, brain stem, and spinal cord, with more variability during patients’ life [17]. The cause of death in AT is often pneumonia or chronic lung disease [18], which might result from defects in chewing and swallowing owing to progressive neurological impairment [19]. Chronic lung disease develops in more than 25% of people with A-T and sometimes immune dysregulation can lead to recurrent pneumonia and bronchiectasis. Moreover, liver disease may emerge over years in A-T individuals and, recently, a systematic study displayed that liver disease is present in the majority of older A-T patients. Structural changes and fibrosis are frequent findings and progress of liver disease is concomitant to neurological deterioration [20]. Accordingly to a recent study, liver disease in A-T tends to get worse as patients become older [21]. This complex clinical condition is worsened by the presence of a wide range of other features like immunodeficiency, growth retardation, and genomic instability, leading to cancer susceptibility, hypersensitivity to ionizing radiation, and sterility [7,22,23]. Moreover, recent studies described cognitive impairments in A-T with some features strictly associated with cerebellar impairment and others mostly related to other brain areas. In particular, early cognitive defects in A-T overlap with cerebellar pathology and are limited to visual–spatial functions. Widespread and deeper cognitive deficits manifest later during the disease progression, when other brain areas are involved, impacting executive function, spatial skills, affect, and social cognition [24,25]. Despite substantial individual variability, the nature of disease progression is unclear as no longitudinal studies have been published. Neurological impairments are worse in older patients, with functional domains in the brain differentially affected [26]. A recent study displayed a non-linear progression of individual patients’ neurological scores [27], which may be linked to the high molecular and cellular complexity as well as to the individual variability and methodological limitations.

The symptomatology is wide owing to the extensive cellular phenotype [28]. Preclinical studies indicate that neurons established from the cerebellum of A-T animal models show not only disrupted network synchronization, but also deep alterations in the non-neuronal system, as found by the study of the astrocytic circuitry [29]. Importantly, chimeric circuitries composed of wild-type astrocytes and *Atm*^−/−^ neurons are indistinguishable from wild-type cultures [29], suggesting that not only neuronal ATM, but also astrocytic ATM, can impact neuronal circuits at levels ranging from synaptic expression to global dynamics. 

The intense research efforts directed at unveiling ATM functions show that an exhaustive comprehension of the ATM-dependent pathways is necessary not only for a deeper understanding of A-T pathophysiology, but also for the increasing number of neurological diseases in which ATM has been recently included.

### ATM Protein

ATM is a serine/threonine protein kinase of approximately 350 kDa belonging to the evolutionary conserved superfamily of phosphatidylinositol 3-kinase-related kinases (PIKKs), also including ataxia-telangiectasia and Rad3-related (ATR) and DNA-dependent protein kinase catalytic subunit (DNA-PKcs). All members of the PIKK family have a kinase domain (KD) at the C-terminal region of the protein, which shares significant homology with the catalytic domain of phosphatidylinositol 3-kinase (PI3K) [30]. In addition to the KD domain, ATM contains other characteristic structural modules: an N-terminal substrate-binding domain (HEAT repeat domain), which binds to some known substrates such as NBS1, p53, and BRCA1 and whose deletion inactivates the protein [23]; a FAT domain, which interacts with ATM’s kinase domain to stabilize the C-terminus region of the protein itself; the PIKK-regulatory domain (PRD); and an extreme C-terminal FATC domain that represents an ordinary carboxy-terminal amino-acid sequence usually located near the termini of the protein [31,32]. These last two domains regulate the KD.

The overall shape of ATM is very similar to that of DNA-PKcs, composed of a head and a long arm [33]. In 2003, Llorca and colleagues first purified human recombinant ATM protein and ATM–DNA bound complex by single-particle electron microscopy and obtained three-dimensional reconstructions showing that DNA binding to ATM induces a large conformational movement of its arm domain towards the head to form a ring-like structure. This ‘ring’ may help to maintain its interaction with the bound linear DNA [34]. These observations are consistent with ATM owning intrinsic DNA binding activity, indispensable for the ATM fundamental role in DNA damage response (DDR).

## 2. ATM Governs Genomic Integrity

### 2.1. ATM Mediates DDR 

**Figure 1:**
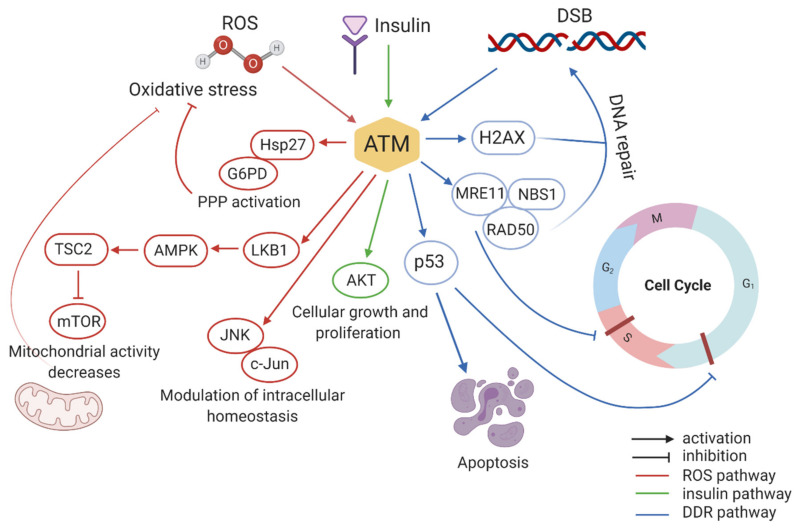
Selected summary of the ATM signalling network. A non-comprehensive schematic of the different ataxia-telangiectasia mutated (ATM) signalling pathways described in the text.

The best-known function of ATM kinase in relation to the phenotypes present in A-T is the response to DNA damage. This protein is at the peak of a signalling cascade that responds to DNA double-stranded breaks (DSBs) and is required to coordinate the resulting cellular responses as cell cycle-arrest, DNA repair, or apoptosis (Figure 1). ATM exhibits increased kinase activity in response to ionizing radiation (IR) or to the radiomimetic compound neocarzinostatin, but not to UV irradiation [35,36,37]. UV irradiation induces relatively less significant DNA damage, whereas IR usually produces severe DSBs inside the genome. Coherently, hypersensitivity to IR (X-rays and γ-rays) was reported in A-T patients after radiotherapy for cancer [38] and in A-T cell cultures [37]. ATM is also necessary for processing the physiological DNA strand breaks that happen during meiosis, immune system maturation, and for telomere maintenance. DNA damage strongly activates ATM in an MRN (Mre11-Rad50-Nbs1)-dependent manner. The importance of the association between ATM and the MRN complex is unveiled by the similarity of three other disorders related to A-T: Nijmegen breakage syndrome by NBS-1 mutants, Nijmegen breakage syndrome-like disorder characterized by Rad50-deficiency, and A-T-like disorder (ATLD) by MRE11 mutants [39,40,41]. These two pathologies differ from A-T, although they share many close clinical symptoms and cellular hallmarks. Upon detection of DSBs, the MRN complex is assembled at the break site and acts as an adaptor for ATM’s auto-phosphorylation at Serine 1981 in human (S1987 in mouse). This phosphorylation is fundamental to obtain the dissociation of the non-functioning ATM dimer into two active monomers [42,43]. Once activated, ATM can phosphorylate downstream DDR targets, among which is the histone variant H2AX, present within chromatin, that is converted into γH2AX by ATM dependent phosphorylation. This seems to be the initial signal for subsequent accumulation of DDR proteins including BRAC1 (breast cancer susceptibility protein-1), MDC1 (mediator of DNA-damage checkpoint protein), and 53BP1 (p53-binding protein), which, together with the multiprotein complex previously formed, facilitate the cellular response to DSBs [44]. Through 53BP1, ATM is able to phosphorylate KRAB-associated protein 1 (KAP-1), which causes chromatin relaxation that provides accessibility for repair factors [45]. ATM can also directly affect DNA repair, for example, phosphorylating the tyrosyl phosphodiesterase 1 (TDP1), which cleaves stalled topoisomerase 1–DNA complexes during transcription and DNA repair [46]. Given the important role of ATM in DNA repair, DNA-damage accumulation may contribute to A-T [11]. Efficient DNA repair is required during development in the thymus, the ovary, the testis, and in particular in the nervous system. As a consequence, human pathologies and mouse models with mutations in DNA-repair enzymes, which are characterized by a neuropathology similar to A-T, show defects in neurogenesis [47,48]. Finally, it is of note that an increased number of human diseases result from defective DNA repair. As A-T, there are Nijmegen breakage syndrome (NBS), Bloom syndrome (BS), and A-T-like disorder, all of which are characterized by chromosomal instability, neurodegeneration, oxidative stress, and immunodeficiency [11]. DNA damage, cell-cycling abnormalities, cerebellar pathology, tauopathy linked to the production of Reactive Oxygen Species (ROS), and alterations in epigenetic pathways have also been demonstrated in the Western Pacific Amyotrophic Lateral Sclerosis and Parkinsonism-dementia Complex (ALS/PDC) syndrome [49]. Accordingly, in a recent study, TDP-43, which has been shown to participate in DNA repair pathways, has been found in the tau-dominated polyproteinopathy of Western Pacific ALS/PDC [50], in particular in the spinal cord, limbic, and cortical regions of patients. Thus, proteins as ATM are acquiring increasing interest and attention also as potential pharmacological targets, as their activity impacts multiple mechanisms at different stages of cells’ life.

### 2.2. ATM Regulates Cell Cycle and Apoptosis 

ATM activation upon DSBs also promotes cell cycle arrest through the activation of cell-cycle checkpoints, which prevents replication of damaged genomic DNA and allows DNA repair (Figure 1) [51,52]. Because several ATM substrates are key effectors of the cell cycle, cells from A-T patients display defective cell-cycle checkpoints [53,54,55], resulting in genomic instability and cancer predisposition [56]. ATM protein is directly involved in cell cycle arrest at both G1 and S phases. The tumour suppressor protein p53 is required for the G1 arrest and its phosphorylation is mediated by ATM on serine 15 after detection of DNA damage. Phosphorylated p53 acts as a transcription factor that activates the production of p21 (also known as CIP1 or WAF1), which in turn inhibits cyclin E and its partner CDK2 that together form a complex required for progression of the cell cycle from G1 to S phase [57]. ATM also plays a key role in cell cycle arrest at S phase, in which DNA replication starts. In fact, one of the first and most important abnormalities that characterizes ATM-deficient cells is a failure to arrest DNA synthesis after ionizing radiation. Upon DSBs, activated ATM phosphorylates NBS1 on serine 343. As a result of NBS1 phosphorylation, the new DNA synthesis site (Replicon) will be inhibited and the cell will be stopped at the S-phase [58]. Alternatively, depending on the cell type and levels of genome damage, ATM can induce apoptosis through p53 and Chk2. This mechanism acquires important relevance in neurons, where the elimination of damaged progenitor cells guarantees the genomic integrity of the developing tissue. In ATM-null mice, the apoptosis pathway is not properly activated, leading to the incorporation of damaged cells into the nervous system [59,60,61,62]. Through all these pathways, ATM helps in preventing neurodegeneration, immune deficiency, sterility, and cancer.

As an example, cell cycle reactivation in adult neurons is an early hallmark of neurodegeneration and CNS injury, and the presence of cell cycle events in the affected neurons is likely to be involved in the development of the disease. Oxidative stress, which is considered to play an important role in the aging and pathogenesis of many neurodegenerative diseases, can induce DNA damage and stimulate cell cycle re-entry by neuronal cells. Although neurons are believed to have permanently blocked their capacity to proliferate once they are differentiated, typically being found in a quiescent state in the adult nervous system, a number of genes that encode for regulators of G1/S transition can be detected in different structures of the normal adult brain. Although cell cycle reactivation has been classically linked to apoptosis [63], cumulative evidence indicates that neurons can actively re-enter the cell cycle, replicate its DNA, and survive during the course of different neurodegenerative diseases [64,65].

### 2.3. ATM and Cancer Susceptibility

Cancer is linked to genomic instability and an increased risk to develop a malignant disease is an important aspect of A-T, so that the diagnosis of some tumors may precede the diagnosis of A-T. About 15% of A-T patients develop malignancy in childhood and, in particular, tumours with lymphoid origin, such as B cell non-Hodgkin lymphoma, T cell lymphoid tumours (T cell lymphoma and T cell acute lymphoblastic leukaemia), and Hodgkin disease. ATM mutations have been described in sporadic T cell prolymphocytic leukaemia [66,67] and B cell chronic lymphocytic leukaemia [68,69]. On the contrary, the impact of ATM mutations on other cancer susceptibility is still controversial, as for breast cancer, as evidenced by data regarding the spectrum and frequency distribution of ATM mutations in breast cancer. As an example, ATM 7271T > G has been considered a rare event in familial breast cancer, whereas ATM IVS10-6T > G mutation did not confer a significantly increased risk of breast cancer. Thus, mutations in *ATM* result in a moderate risk of cancer and a recent multicentre case-control study includes for ATM some genetic variants associated with an increased risk of breast cancer [70]. In conclusion, the hypothesis is that the risk may be higher for certain mutations, including missense mutations responsible for abnormal ATM protein, thus involvement of ATM gene mutations in breast cancer may be crucial in particular families.

## 3. Multiple Roles of ATM Protein Kinase: Beyond the DNA Damage Response

**Figure 2:**
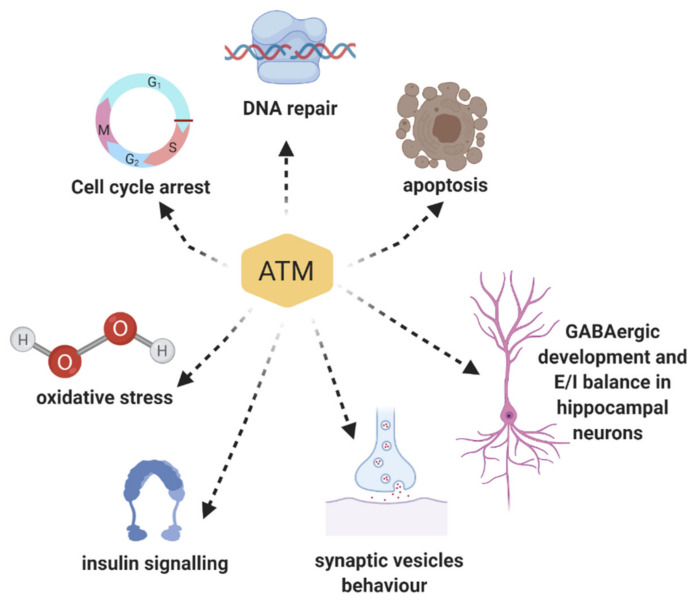
Scheme of the different roles of ATM. ATM plays different roles both in the nucleus and cytoplasm.

ATM’s involvement in DDR clearly explains many pathological hallmarks of A-T disease including cancer predisposition, hypersensitivity to ionizing radiation, immunodeficiency, and infertility [1]. However, neurological symptoms such as ataxia, speech defects, abnormal body movements, and learning impairments are still difficult to explain. Indeed, considering the CNS phenotype of the ATM-mutant brain, we must conclude that the related pathological mechanism is certainly more complex than a deficit associated only with the DNA repair mechanism [9]. Furthermore, without ATM, cells can still repair the DNA, but through a more time-consuming processes [71,72]. Nonetheless, this delay and the enhanced stress related do not seem enough to explain the neurological phenotype in A-T. One possible explanation is that ATM plays additional roles in cells of developing and mature CNS in different districts. In line with this hypothesis, many studies revealed the presence of ATM in the cytoplasm, and not only in the nucleus as previously described, playing a different, but still important role, than DDR (Figure 2). Thus, while the main presence/function of ATM in the molecular pathways following DNA damage has been explored in depth, many recent studies have brought to light ATM’s involvement in a wider spectrum of cellular activities, especially in neurons [73].

### 3.1. ATM Functions in Oxidative Stress 

Oxidative stress is defined as a disruption of the balance between the production of reactive oxygen species (free radicals) and antioxidant defenses [74]. Most of the DNA-damages are owing to normal metabolic by-product and not to IR or exogenous radiomimetic chemicals. Indeed, oxidative stress produced by endogenous metabolism causes, at least in part, the constitutive low-level DNA damage response often detected in cultured cells [75]. Because of their intense metabolic activity, neurons are subjected to a considerable oxidative stress, and the deregulation of these processes has been associated with various neurodegenerative conditions [76]. There is consistent evidence that oxidative stress contributes to the A-T phenotype. Accordingly, oxidative stress has been reported in patients with A-T, in A-T cell cultures, and in brain and tissues from ATM-deficient mice [77,78]. ATM-deficient cells are hypersensitive to oxidative damage and radical scavengers can alleviate this sensitivity [79]. Reliene et al. [80] demonstrated that *Atm*^−/−^ mice show an increased frequency of DNA deletions and higher levels of 8-OHdeoxyguanosine, a marker of oxidative DNA damage. When subjected to a diet supplemented with the antioxidant NAC (N-acetyl-l-cysteine), the genetic instabilities revert to normal levels, suggesting the impact of oxidative stress on DNA damage and confirming the involvement of ATM in oxidative stress signalling. Not surprisingly, neuronal oxidative stress has been suggested as a key player in the neurodegeneration processes occurring in A-T [81,82]. In a pivotal study, Guo and colleagues revealed that oxidation can directly induce ATM activation in the absence of DSBs [83]. Whereas after DNA breaks, S1981 phosphorylation of ATM leads to ATM activation and dimer dissociation, under oxidation, disulphide bonds are formed, resulting in covalent linkages between the subunits of the dimer. In this configuration, kinase activity is found to be activated. Surprisingly, this activity is independent of S1981 state of phosphorylation and MRN complex. Taking advantage of mutational studies, Guo et al. demonstrated that the presence of cysteine-2991 in the C-terminus of ATM is crucial for this activation via oxidation [83]. In this condition, ATM acts as an oxidative sensor, directly controlling the redox homeostasis of the cell through different pathways (Figure 1). It has been demonstrated that ATM maintains redox homeostasis by regulating central carbon metabolism. Under oxidative stress conditions, ATM re-routes the metabolic flux from glycolysis to the pentose phosphate pathway (PPP) by promoting the phosphorylation of Hsp27 that in turn binds to glucose-6-phosphate dehydrogenase (G6PD), stimulating its activity [84,85]. PPP is the principal responsible for the production of NADH in the cell. Promoting PPP function, ATM increases NADH production, which protects cells against ROS toxicity, as it is a cofactor for many antioxidant enzymes such as glutathione reductase. Under oxidative stress conditions, ATM also represses mTOR via activation of the LKB1/AMPK pathway. By inhibiting mTOR, ATM reduces mitochondrial activity and ROS levels, thus protecting cells from oxidative stress [86,87]. Mitochondrial dysfunction and its relationship with neurological defects in rat neurons, worms (C. elegans), and mouse models of A-T have been recently assessed [88]. Here, restoration of the NAD^+^/SIRT1 signalling by NAD^+^ supplementation reduces the severity of neurological defects and increases the healthspan and lifespan in these systems. NAD^+^ replenishment also stimulates DNA repair and mitophagy in A-T models, indicating that ATM affects NAD^+^ regulation of mitophagy rather than DNA repair.

**Figure 3:**
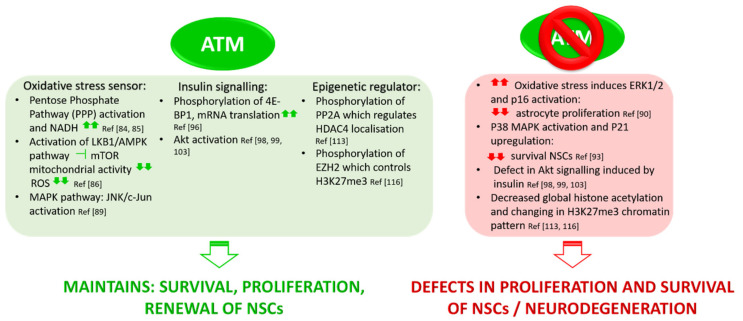
ATM maintains NSCs’ survival and protects from neurodegeneration. Through its roles in the maintenance of oxidative stress homeostasis, insulin signalling pathways, and epigenetic regulation, ATM is involved in survival, proliferation, and renewal of neuronal stem cells (NSCs). In ATM deficiency, neural progenitors show a reduced survival rate and defects in proliferation and differentiation. ATM absence also affects the proliferation of astrocytes, which in turn fail to support neuronal homeostasis, leading to neurodegeneration.

Several studies demonstrated ATM’s role in preventing oxidative stress via the MAPK pathway. Lee et al. showed that ATM modulates intracellular redox homeostasis by phosphorylating c-Jun on Ser63 and Ser73 residues via JNK [89]. Kim and Wong demonstrated that in *Atm*^−/−^ astrocytes ERK1/2 (extracellular signal-regulated protein kinase 1 and 2) is constitutively activated owing to high levels of ROS. This mediates the upregulation of cyclin dependent kinase inhibitor (p16), which in turn reduces the proliferation of astrocytes. Through this pathway, ATM contributes to the proper growth, maturation, and homeostasis of astrocytes, which consequently protect neurons under oxidative stress conditions. Liu et al. demonstrated that *Atm*-deficient astrocytes show increased levels of oxidative stress markers such as MnSOD, Cu/Zn SOD, and Hsp70, and an upregulation of spontaneous DNA synthesis, endoplasmic reticulum (ER) stress, and redox-sensitive phosphorylation of ERK1/2 [90]. In this condition, *Atm*^−/−^ astrocytes cannot provide antioxidant support to neurons, probably resulting in neurodegeneration or neuronal cell death, which is one of the major features of A-T disease. Because of its role in maintaining the redox homeostasis of the cell, several studies suggested that ATM is required for maintaining neural stem cell survival, proliferation, and differentiation [91,92,93] (Figure 3). Kim et al. demonstrated that neuronal stem cells (NSCs) from neonatal subventricular zone (SVZ) tissues of *Atm*^−/−^ mice display defective self-renewal and proliferation. The underlying molecular mechanism involves p38 MAPK activation and polycomb protein Bmi-1 downregulation [93]. Bmi-1 is crucial for NSC self-renewal and survival as it silences the genes encoding for cell cycle inhibitor p21 and is stabilized by Akt, which prevents its proteasomal degradation. In *Atm* deficient NSC, increased oxidative stress activates the p38 MAPK pathway that inhibits Akt dependent Bmi-1 phosphorylation, destabilizing the protein. Bmi-1 degradation promotes p21 upregulation, causing a decrease in survival and proliferation of NSCs.

Together, these studies highlight the importance of ATM in controlling the redox homeostasis of the cell. This acquires relevance in neurons where ATM deficiency impacts oxidative stress signalling pathways with deep consequences on neural stem cells proliferation, neurodegeneration, and aging (see Section 4).

### 3.2. Role of ATM in Insulin Signalling Pathways 

Another key role of ATM, impacting A-T phenotype, is its involvement in insulin signalling pathways (Figure 1). Insulin-like growth factors play a fundamental role in neuronal survival, cell metabolism, and human growth [94]. Abnormalities in these pathways may provide a basis for understanding aspects of A-T that cannot be ascribed only to DDR defects, such as growth abnormalities, insulin resistance, altered cognition, and neurodegeneration. Insulin and insulin-like growth factors activate mTORC2 and lead to the subsequent activation of Akt (protein kinase B). Akt phosphorylates TSC2, which releases mTORC1 inhibition, allowing the phosphorylation of 4EBP-1 (elongation factor 4E binding protein) and ribosomal S6 kinase. Phosphorylated 4E-BP1 releases eIF4E for the initiation of mRNA translation and protein synthesis [95]. Yang and Kastan demonstrated that ATM kinase activity increases threefold in response to insulin in rat 3T3-L1 cells that have been differentiated into adipocytes [96]. They showed that the phosphorylation of 4E-binding protein 1 (an insulin-responsive cytoplasmic protein member of a family of translation repressor proteins) at Ser111, a different site from those phosphorylated by mTORC1, is mediated by ATM and promotes mRNA translation [96]. Thus, cells lacking ATM display a decreased insulin-induced dissociation of 4E-BP1 from eIF-4E with a consequent dysregulation in the insulin-pathway controlling translation initiation. ATM also induces the activation of PKB/Akt in response to insulin or Ɣ-radiations. This effect is mediated by the ATM phosphatidylinositol 3-kinase domain, which directly phosphorylates Akt at Ser473, causing the full activation of this transcription factor. Through the response to insulin and other growth factors, Akt plays a key role in many physiological processes such as protein translation, cell proliferation and survival, and glucose uptake [97]. Coherently, cell lines derived from A-T patients display altered Akt pathways and *Atm*-null mice show a drastic decrease of Akt phosphorylation in Ser473 [98,99]. A-T patients are insulin-resistant, which could be partially explained by the lack of full Akt activity [100]. ATM-dependent activation of Akt was found to be required for cell differentiation [101] and to prevent apoptosis [102]. Interestingly, in a comparison study using SH-SY5Y cells, Li et al. showed that, in undifferentiated cells, ATM is mainly involved in the nuclear response to DNA damage; conversely, in differentiated cells, it mainly mediates Akt signalling to promote cell survival [103]. These data suggest that the lack of Akt signalling in A-T may contribute to neurodegeneration and highlight the link between cytoplasmic ATM and neurological phenotype in A-T. Moreover, preclinical studies revealed that insulin-like growth factor 1 (IGF-1) plays a crucial role in both prenatal and postnatal phases of brain development. It modulates synaptic plasticity, synapse density, neurotransmission, and adult neurogenesis [104,105,106]. During the first period of life, pharmacological approaches and animal manipulations able to upregulate early IGF-1 levels result in a reduced ratio between the expression of two cation-chloride transporters, NKCC1 and KCC2, whose balance is instrumental for maturation of GABAergic neurotransmission [107]. Enhanced IGF-1 is also thought to mediate the induction of hippocampal BDNF, and together they are considered as key factors of the environmental enrichment effect on learning and memory [108]. Thus, on the basis of this evidence and considering that IGF-1 signaling is downregulated in A-T phenotype [109], we can speculate that in, ATM-linked pathology, cognitive alterations and dysfunctions in cerebellum and cerebrum may also be a consequence of a defective insulin pathway.

### 3.3. ATM Mediates Epigenetic Regulation 

By its interaction with HDAC4, a member of class IIa histone deacetylases (HDACs), ATM seems to act as an epigenetic regulator in neurons. It is widely known that class I and class IIa HDACs play important roles in brain development and neuronal survival [110,111]. HDAC4 is abundant in neurons, especially in Purkinje cells, where it is predominantly cytoplasmic. HDAC4-deficient mice show a significant cerebellar atrophy and Purkinje cells display structural and density abnormalities compared with wild-type. HDAC4 localization depends on its phosphorylation state: the dephosphorylated form accumulates in the nucleus, whereas it must be phosphorylated to stay in the cytoplasm [112]. Li J. and colleagues [113] discovered that, in samples from A-T patients and in *Atm*-null mice, HDAC4 in neurons localizes predominantly in the nucleus, concluding that this nuclear accumulation is ATM-dependent. In fact, ATM mediated phosphorylation downregulates the protein phosphatase 2 (PP2A) activity, the HDAC4 phosphatase. In the absence of ATM, the increased activity of PP2A on HDAC4 causes HDAC4 nuclear accumulation. Consequently, in *Atm*-deficient neurons, global histone acetylation is decreased, meaning a closed chromatin configuration and a reduced general transcription. Importantly, HDAC4 directly silences the transcriptional activity of two pro-survival transcription factors: MEF-2 (myocyte enhancer factor 2) and CREB (cAMP response element-binding protein) [114,115]. The authors also observed a decreased transcription of other crucial neuronal genes such as *Bdnf, NR2a,* and *Nrxn*. Thus, the dysregulation of this pathway determines genome-wide alterations in neurons probably contributing to neurodegeneration [113].

The same group reported that, in neurons, histone H3K27 tri-methylation (H3K27me3), mediated by polycomb repressive complex 2 (PRC2), is also ATM-dependent [116]. EZH2 (enhancer of zeste homolog 2), which is a core catalytic component of PRC2, has been found to be a new ATM kinase target: ATM-mediated S734 phosphorylation of EZH2 reduces its stability. In the absence of ATM, PRC2 formation is elevated along with H3K27me3. This change of H3K27me3 chromatin-binding pattern is strictly related to cell-cycle re-entry and cell death of *Atm*-deficient neurons [116]. Furthermore, knockdown of EZH2 rescues Purkinje cell degeneration and behavioural alterations in *Atm*-deficient mice, indicating EZH2 hyperactivity as another key factor in A-T neurodegeneration.

### 3.4. ATM and Neuronal Function: Implication in Synaptic Vesicles’ Behaviour in Neurons 

A very interesting observation about the role of ATM besides DDR concerns its association with cytoplasmic vesicles, particularly with synaptic vesicles in neurons. In 1996, Lakin and colleagues found that ATM co-fractionates and co-localizes with cytoplasmic vesicles in A-T cell lines [117,118]. Two years later, Watters et al. demonstrated that ATM cytoplasmic fraction associates with peroxisomes and endosomes [119] and is required for the functioning of these organelles. More recently, it has been shown that ATM also associates with mitochondria, regulating their function and mitophagy [120,121,122]. Moreover, the cytoplasmic fraction of ATM does not change in amount or in localization in response to IR [118,123], meaning that the ATM cytoplasmic pool is distinct from the nuclear one and is crucial for cellular and sub-cellular activities far from the function in genome surveillance. In a pivotal study, Lim and colleagues demonstrated that cytoplasmic ATM binds β-adaptin, a member of the AP-2 adaptor complex, which is involved in clathrin-mediated endocytosis of receptors, membrane trafficking, and cell signalling [124]. This interaction was demonstrated in vitro, but also in vivo, by co-immunoprecipitation and co-localization studies. The consequence of ATM and β-adaptin interaction is still unclear, but indicates that cytoplasmic ATM may play an important role in intracellular vesicle and/or protein transport mechanisms. In fact, several ATM-related lipid kinases such as Vps34 and PI-3K are crucial in stimulating vesicles and protein transport [125]. This also allows to speculate that cytoplasmic ATM may act in vesicle transport and ATM deficiency could deeply impact protein cellular homeostasis. In this context, surprising discoveries come from studies of cytoplasmic ATM in neurons. In the literature, it is known that ATM is predominantly localized in the nucleus of dividing cells, while in post-mitotic cells like Purkinje and granular neurons in the cerebellum, it has a significant distribution in the cytoplasm [117,126]. This has been confirmed by Karl Herrup’s group [127], which identified cytoplasmic ATM in neuronal cells from brain structures and synaptosome fraction, but not in other peripheral tissue. They showed that neither irradiation nor topoisomerase inhibitors activate cytoplasmic ATM, suggesting that its function is unrelated to DDR. Taking advantage of immunofluorescence and immunoprecipitation experiments, they revealed two novel binding partners of cytoplasmic ATM, VAMP2 and Synapsin-I, known synaptic vesicle proteins that localize in presynaptic nerve terminals. Synapsin-I is a linker protein that keeps synaptic vesicles (SVs) related to the cytoskeleton, especially to actin, in presynaptic terminals [128]. Its function is crucial in maintaining and stabilizing the reserve pool of SVs in the cytoplasm, near the plasma membrane, but it is also essential to make SVs ready to be released. Thus, its main function is the homeostatic regulation of synaptic transmission [129]. Vesicle-associated membrane protein2 (VAMP2; also known as synaptobrevin2), instead, is a central member of the SNARE (soluble N ethylmaleimide-sensitive fusion protein-attachment protein receptor) complex that mediates synaptic vesicle fusion with the cell membrane, allowing the neurotransmitter release [130]. In fact, by means of FM4-64 dye, Li et al. monitored the uptake and release of vesicles from cultured neurons derived from either wild-type or *Atm^tm1Awb^* mice and found that vesicles’ release is reduced in *Atm* deficient cells [127]. Vail et al. showed impaired Long Term Potentiation (LTP) and reduced paired-pulse facilitation in *Atm*^−/−^ mice, demonstrating at which extent the complete loss of ATM impacts neuronal plasticity and synaptic function. With a stochastic optical reconstruction microscopy, they also found that ATM is significantly more closely associated with Piccolo (a presynaptic marker) than with Homer1 (a postsynaptic marker) [131]. In a recent study, Cheng et al. deepened ATM function at the neuronal synapse [132]. Taking advantage of super-resolution microscopy and co-immunoprecipitation experiments, they demonstrated that ATM and ATR in cultured cortical neurons specifically segregate to different classes of vesicles playing complementary roles: ATM with excitatory (VGLUT1+) vesicles and ATR with inhibitory ones (VGAT+). They also demonstrated that the two proteins are in balance with each other, so ATM or ATR deficiency leads to a compensatory increased expression of the other kinase. This compensatory effect seems to occur rapidly and quickly influences the excitatory/inhibitory (E/I) balance of the neuronal network [132].

**Figure 4:**
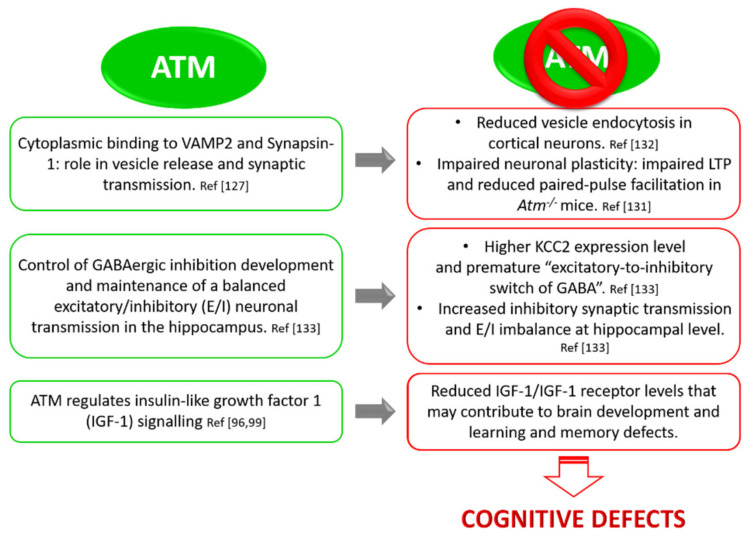
ATM controls synaptic function, preventing cognitive alterations. ATM is involved in synaptic vesicles release and endocytosis, GABAergic development, and maintenance of a balanced excitatory/inhibitory (E/I) neuronal transmission. In ATM deficiency conditions, neurons display impaired synaptic plasticity (LTP), defect in the maturation of inhibition, and E/I imbalance. These defects might underlie the neurological symptoms of A-T.

Together, these studies highlight the importance of cytoplasmic ATM in neuronal function, providing important insights into the neurological symptoms of A-T (Figure 4).

### 3.5. ATM and Neuronal Function: Implication in GABAergic Development and Excitatory/Inhibitory Equilibrium 

In line with the emerging roles of ATM in regulating neuronal functions, Pizzamiglio et al. unveiled a pivotal role of ATM in controlling the development of GABAergic inhibition and in maintaining a balanced excitatory/inhibitory (E/I) neuronal transmission at the hippocampal level [133]. By immunofluorescence and electrophysiological experiments, they showed that primary hippocampal neurons obtained from *Atm* heterozygous (*Atm*^+/−^) mouse embryos display an E/I imbalance towards inhibition indicated by the following: (i) a higher frequency of miniature inhibitory post synaptic currents (mIPSCs); (ii) an increased number of inhibitory synapses; and (iii) GABA-containing synaptic vesicles. They confirmed these results also reducing the amount of ATM in WT hippocampal neurons by the specific siRNA. *Atm*^+/−^ hippocampal cultures displayed a premature “excitatory-to-inhibitory switch of GABA”, a crucial step in the development of GABAergic system. γ-aminobutyric acid (GABA) is the main inhibitory neurotransmitter in the adult mammalian brain; it binds GABA_A_ receptors, which mediate a net influx of chloride with consequent membrane hyperpolarization and reduction of cell firing. Conversely, early during development, the intracellular chloride concentration [Cl^−^]_i_ rises in such a way that the opening of anion channels by GABA produces a chloride efflux and a membrane depolarization able to evoke action potentials [134,135,136]. The mechanisms underlying chloride accumulation inside immature neurons depend on a different efficacy of chloride co-transporters such as NKCC1 and KCC2, which import and export chloride, respectively [137]. Before and immediately after birth, chloride accumulates inside the cell owing to a reduced expression of the cation–chloride exporter KCC2. Later during development, the intracellular chloride concentration decreases thanks to the up-regulation of KCC2 during the “excitatory-to-inhibitory switch of GABA” [138]. This phenomenon is fundamental for the development of the GABAergic network because it is widely accepted that it regulates the number and the strength of inhibitory synapses. In particular, an anticipated GABA switch is known to increase GABAergic synapses and the frequency of GABAergic miniature postsynaptic currents [139,140]. Taking advantage of calcium imaging experiments, Pizzamiglio et al. demonstrated that *Atm*^+/−^ hippocampal neurons show an accelerated switch of GABA, related to higher KCC2 expression in hippocampal tissues. These rearrangements seem to depend on the increase in phosphorylated ERK and on the lower expression of its phosphatase, PP1 [133]. ERK activation could sustain the higher KCC2 expression through the Egr4-dependent activation of the *Kcc2b* promoter [141]. These data demonstrate that alterations of ATM expression impact the development and function of GABAergic system, leading to an E/I imbalance in favour of inhibition. Notably, proper GABAergic transmission and E/I balance are necessary for correct brain maturation, from neuronal proliferation, migration, and differentiation to experience-dependent organization and plasticity of local circuits [142,143,144,145]. Thus, this study provides a rationale for the cognitive defects described in A-T and links ATM among those proteins possibly involved in neuropsychiatric disorders characterized by altered E/I balance and inhibitory system.

## 4. ATM Dysregulation in Neurological Diseases 

Through its multiple functions in both the nucleus and cytoplasm, ATM plays a fundamental role in neuronal homeostasis; accordingly, neurological defects are key aspects of A-T phenotype. Several studies revealed ATM involvement in neurological conditions beyond A-T (Table 1), highlighting ATM as a new potential pharmacological target in brain pathologies.

### 4.1. ATM Involvement in Neurodegenerative Disorders and Brain Senescence

Neurodegeneration is a classic hallmark of A-T. As previously described, A-T is characterized by progressive cerebellar neurodegeneration and loss of Purkinje and granular cells [146,147]. Although the clear molecular mechanism is still debated, neurodegeneration in A-T has been linked to defective DNA damage and repair in brain, dysregulation of redox homeostasis, the role of ATM in suppressing cell cycle re-entry in post-mitotic neurons, and a possible role in neuronal stem cells survival and differentiation [17,32,148,149].

Many neurodegenerative diseases are associated with a high degree of oxidative stress, elevated DNA damage, defective DNA repair efficiency, and dysregulation of proteins involved in DNA damage response and cell cycle, among which is ATM [150]. Petersen et al. demonstrated that even a partial reduction of ATM kinase activity is enough to cause neurodegeneration in Drosophila [151]. They showed that one of the mechanisms underlying neuronal death involves glial cells and inflammation, suggesting that early-onset CNS neurodegeneration in A-T is similar to late-onset CNS neurodegeneration in diseases such as Alzheimer’s and Parkinson’s. Indeed, the prolonged activation of microglia, the resident innate immune cells in the CNS, in these neurodegenerative pathologies is thought to drive neurotoxicity. For example, in Alzheimer’s disease, amyloid-β is responsible for the prolonged activation of microglial cells, which then release neurotoxic factors such as TNF-α and reactive oxygen species. Moreover, in Parkinson disease, the overactive microglia produce reactive oxygen species in response to damaged dopaminergic neurons. Thus, Petersen et al. revealed a further correlation between neurodegeneration and the innate immune response exploiting the fly model of the human neurodegenerative disease A-T.

In line with these findings, Shen et al. recently demonstrated that decreased ATM activity is involved in the mechanism of neuronal death found in Alzheimer’s disease (AD), the most common form of dementia globally. They showed reduced ATM levels in lysates of human frontal cortex and cerebellum of Alzheimer’s disease (AD) subjects. They also suggest that, in AD, ATM activity in neurons is low, as indicated by three indirect measurements of ATM signalling including (i) the nuclear translocation of histone deacetylase 4 (HDAC4); (ii) the trimethylation of histone H3; and (iii) the appearance of cell cycle proteins, specifically cyclin A2 [152]. Analysing different brain areas involved in AD, they showed that these same neurons present reduced ATM protein levels and, even if they represent only a fraction of the total neurons in each affected region, their number significantly correlates with disease stage. Moreover, using the localization of HDAC4 as a reporter of ATM signalling, they showed in three different transgenic models of AD that ATM activity in neurons is reduced in both the hippocampus and frontal cortex, confirming that it is central to the neuronal death mechanism [152]. Thus, while genetic ATM deficiency leads to an early childhood neurodegenerative condition, the sporadic loss of ATM function in individual neurons can occur later in life in association with AD. The resulting ATM deficiency may contribute to the mechanism leading to loss of neuronal cell cycle control and, ultimately, to cell death. The suggestion that emerges from this study is that enhancing the level of ATM, especially in the brain, would be worth exploring as a novel therapeutic approach in AD.

Dysregulation of ATM signalling has also been addressed to Huntington’s disease (HD), a genetic neurodegenerative disorder caused by a CAG-repeat expansion in the first exon of the HTT gene encoding the huntingtin protein. Unlike A-T and AD, where ATM function is absent or reduced, respectively, in HD, it is the increased level of ATM that correlates with the disease progression. Cells transfected with the mutant form of HTT (mHTT) show elevated DNA damage and ATM activation [153,154]. In a pivotal study, Lu and colleagues revealed that ATM signalling is consistently elevated in cells derived from the HD mouse model (BACHD) and in brain tissue from HD mice and patients [155]. They did not clarify the molecular mechanism underlying this aberrant ATM activity, but they demonstrated that blocking ATM with a specific shRNA ameliorates N-terminal mHTT fragment toxicity in cells and Drosophila HD models. Impressive results were obtained when they crossed mice heterozygous for an *Atm* null allele (*Atm*^+/−^) with BACHD mouse model of HD. They showed that reducing *Atm* gene dosage (i) ameliorates behavioural deficits such as spontaneous locomotion, motor coordination, and depressive-like behaviours in the forced swimming test and (ii) reduces HTT aggregates in cingulate cortex and striatal atrophy [155]. Thus, blocking ATM kinase activity could provide a novel clinical intervention to treat HD [156]. In a recent study, Maiuri et al. clarified the possible mechanism liking ATM function and huntingtin [157]. Taking advantage of super-resolution microscopy and biochemical assays, they revealed that huntingtin co-localizes and scaffolds proteins of the DNA damage response pathway in response to oxidative stress and its localization to DNA damage sites depends on ATM kinase activity [157]. They speculated that ATM inhibition is beneficial as it blocks the recruitment of mutant huntingtin to the damage site, which may slow down or impair the DNA repair process.

In addition to neurodegenerative disorders, ATM is also involved in brain senescence. Progressive accumulation of DNA damage and oxidative stress are causally involved in cellular senescence and organism aging [158]. Through its cofactor ATMIN, ATM protects against oxidative stress and DNA damage in the aging brain [159]. Notably, in a very recent study, Kreis and colleagues demonstrated that, in oxidative stress conditions, ATM phosphorylation of the actin-binding protein drebrin (DBN) improves stress resilience in dendritic spines [160]. DBN is a conserved F-actin side-binding protein that reduces actin filament turnover [161]. It is enriched in dendritic spines, where it controls spine morphology and function, and its progressive loss has been linked to cognitive defects associated with aging [162]. In persistent oxidative stress condition and aging, ATM is activated and phosphorylates DBN, resulting in the stabilization of the protein. Subsequently, DBN safeguards against ageing-induced dendritic spine degeneration, early synaptic dysfunction, and cognitive decline [160]. During aging, a progressive decline in ATM-centered DNA repair machinery was shown, but a recent study established a direct causal link between DNA repair machinery and longevity, revealing a pro-longevity role of ATM. Quian and colleagues [163] demonstrated that low doses of chloroquine (CQ) activate ATM, resulting in the pro-longevity SIRT6 deacetylase stabilization, reached by the prevention of SIRT6 ubiquitination and proteasomal degradation. The authors showed that CQ administration globally ameliorates coordination performances as increased running endurance in mice besides inhibited glycolysis, lowered serum lactate levels, and attenuated body weight decline, resulting in a longer lifespan.

Taken together, these studies confirm that ATM protein is so crucial in the homeostasis of the central nervous system that any alterations of its levels and signalling are linked to cellular dysfunction and pathology.

### 4.2. ATM Involvement in Cognition

Cognition is the mental process through which we acquire knowledge, and we understand through thought, experience, and senses. It includes different aspects of intellectual functions and processes such as attention, memory, judgment and evaluation, reasoning, decision making, comprehension, and language.

As previously described, even if the main neurological defects in A-T consist of progressive cerebellar ataxia and in symptoms involving extrapyramidal pathways, a cognitive phenotype is also described. It is widely accepted that the cerebellum participates in the organization of higher order functions. In fact, the cerebellar cognitive affected syndrome (CCAS) is characterized by deficits in executive function, linguistic processing, working memory, and spatial cognition, resulting in intellectual disability [24,25,164]. Cognitive impairments have been reported in children with acquired or congenital cerebellar disorders [165] and developmental, intellectual, and behavioural defects have been associated with complex cerebro-cerebellar childhood disorders and neuropsychiatric disorders, including autism [24,25,164,166,167]. The involvement of pure cerebellar, non-cerebellar, or cerebro-celebellar circuits and the time of onset of the disorder influence the functional outcome, with a greater impact in early stages of development. This is the case of A-T, in which *ATM* mutations affects the CNS since the early embryonic stage. In a preliminary study, Hoche and colleagues found cognitive and behavioural symptoms characteristic of the CCAS in children with A-T. Despite that these defects could be entirely ascribed to the cerebellar syndrome, patients also display defects in attention, late learning, and memory that cannot be exclusively ascribed to the cerebellum [24]. In this regard, in a study from Volkow and colleagues [168], both *ATM* homozygotes and heterozygotes patients showed reduced glucose metabolism in different brain areas, including the hippocampus, the brain structure fundamental for memory and learning. This suggests that mutations in *ATM* gene impact not only cerebellar functions, but also properties of different brain regions important for cognition. In addition, Pizzamiglio and colleagues demonstrated that reduced ATM expression leads to an E/I imbalance in favour of inhibition in the hippocampus, a process that could support the progressive decline in cognitive performances observed in clinic [133].

Moreover, Mostofsky and colleagues [169] showed a significantly reduced verbal IQ and considerable problems with judgment of duration in 17 A-T patients. The IQ of younger patients, tested with a non-verbal test, seems to fall into the average (IQ = 100), while that of older patients fits in values between 57 and 83 [170]. Moreover, Hoche and Colleagues [25] found full-scale IQ scores impaired especially in school children and adolescents/young A-T patients rather than in toddlers and pre-schoolers, where cognitive changes were limited to mild visual–spatial disorganization.

These data suggest that cognitive defects in A-T at younger ages are mild and limited to the cerebellar pathology, with widespread cognitive impairments emerging with age and disease progression. Recent studies revealed that exposure of mice to physiological learning behaviours determined the formation of “activity-induced DSBs” [171,172] restricted to few loci through the entire genome. These loci are enriched for the early response genes *Fos, Npas4, Egr1,* and *Nr4a1* [140] and their rapid expression is crucial for experience-driven changes to synapses, highlighting the importance of DSBs in shaping synaptic plasticity and in controlling neuronal function and behaviour. Considering the crucial role of activity-dependent genes expression in neurons, defects in DNA repair have important pathophysiological implications. In facts, loss-of-function mutations in different proteins implicated in DDR generate devastating neurological disorders besides A-T, such as ATR-Seckel syndrome, a complex pathology generated by mutations in *ATR* gene and characterized by microcephaly and progressive aging, LIG4 syndrome, ATLD (A-T-like disorder), and Nijmegen breakage syndrome [173].

About that, many studies have shown altered DNA damage and DNA repair in both patients and mouse models of AD [174,175]. Besides neurodegeneration, AD is characterized by a gradual cognitive decline with recent memory impairment, language disorders and alterations of abstract reasoning, concentration, and thought sequencing [176]. Recently, Suberbielle and colleagues showed that human amyloid precursor protein (hAPP), a well described mouse model of AD, displays increased neuronal DSBs at baseline and more severe and prolonged activity-induced DSBs after exploration [171]. Moreover, the same group found that BRCA1, a tumour suppressor protein indispensable for DNA repair, is reduced in AD patient’s brains and hAPP transgenic mice [177]. They demonstrated that knocking down neuronal BRCA1 in the dentate gyrus of wild-type mice causes increased DSBs, neuronal shrinkage, synaptic plasticity deficiencies, and learning and memory defects, indicating that this protein protects the neuronal genome, and critically supports neuronal integrity and cognitive functions. Moreover, the pathological accumulation of Aβ depletes neuronal BRCA1, which may contribute to cognitive impairment in AD [177]. Moreover, failure in ATM signalling and the consequent reduction of DDR efficiency in both the AD mouse model and human AD tissues [152] suggest that altered DDR could be an important contributor not only to neurodegeneration in AD, but also to cognitive decline.

Together, these findings highlight how DDR, and in particular ATM protein, plays a fundamental role in cognitive decline not only in A-T, opening the way to new possible pharmacological targets for cognitive impairments.

## 5. Conclusions and Future Perspectives

A-T is a rare multisystem genetic disorder characterized by severe neurological symptoms and important complications, among which are a compromised immune system, progressive respiratory failure, and increased risk of malignancies. Unfortunately, there is no cure for A-T and, given the complexity and severity of the disorder, a multidisciplinary and polytherapy approach is necessary. Therapy improves the patient’s quality of life, but unfortunately remains only supportive and does not change the poor prognosis of the disease.

Long-term survival of classic A-T patients is accompanied by an increase in both physical and psychosocial problems, suggesting that ATM impairments affect not only structures typically involved in the pathology and that additional information on ATM functions is still missing. Especially, the impact of ATM dysfunctions in the central nervous system and particularly in non-cerebellar neurons is only marginally addressed. For example, cognitive functions are formally tested only in few patients and, in some of them, cognitive alterations are present. Moreover, there is the possibility that problems in cognition are partially underestimated owing to motor and speech impairments. For sure, cognitive dysfunction and mood disturbances can be part of the cerebellar cognitive affective syndrome, but here, we review past and recent results on multiple ATM roles on the basis of which the brain pathological phenotype of A-T appears to be more complex. The current attempt to decipher ATM’s functions in nucleus and cytoplasm as well as in non-cerebellar areas can help researchers better understand (i) the pathogenesis of A-T, (ii) additional ATM-related diseases, and (iii) possible ATM-based therapeutic approaches. As an example, current cancer treatment strategy is based on radiation and genotoxic drugs. In this context, inhibitors of ATM and other DNA repair proteins have been studied to offer a chance to reduce the radio- or chemo-resistance caused by DNA repair mechanisms. Moreover, owing to the multiple roles of ATM in multiple brain regions, the fine-tuning of ATM may be developed as a new pharmacological approach for the treatment of neurological conditions including cognitive deficits.

## Figures and Tables

**Table 1:** Alteration of ataxia-telangiectasia mutated (ATM) expression levels in neurological conditions beyond A-T.

References	Experimental Models	ATM Levels	Changes
Petersen et al. [151]	• Drosophila mutant for *ATM*	Low	• Neuron and glial cell death in the adult brain • High inflammation • Reduction in mobility and longevity
Shen et al. [152]	• R1.40, PS/APP, 3xTg mouse models of AD • Human AD brains	Low	• Nuclear translocation ofhistone deacetylase 4 • Trimethylation of histone H3 • The presence of cell cycle activity • Low ATM signaling in neurons in regions where degeneration is prevalent
Illuzzi et al. [153] Giuliano et al. [154]	• Cells transfected with mHTT	High (activation)	• Elevated DNA damage and oxidative stress
Lu et al. [155]	• BACHA mouse model of HD • Human HD brain	High	Reducing *Atm* ameliorates: • mHTT fragment toxicity in cells • spontaneous locomotion, motor coordination, depressive-like behaviours
